# Non-Isothermal Crystallization Kinetics of Short Glass Fiber Reinforced Poly (Ether Ether Ketone) Composites

**DOI:** 10.3390/ma11112094

**Published:** 2018-10-25

**Authors:** Xujing Yang, Yazhuo Wu, Kai Wei, Wenjun Fang, Haofei Sun

**Affiliations:** State Key Laboratory of Advanced Design and Manufacturing for Vehicle Body, College of Mechanical and Vehicle Engineering, Hunan University, Changsha 410082, China; yangxujing@hnu.edu.cn (X.Y.); wuyazhuo@hnu.edu.cn (Y.W.); wenjunfang@hnu.edu.cn (W.F.); sunhaofei@hnu.edu.cn (H.S.)

**Keywords:** poly (ether ether ketone), short glass fiber, non-isothermal crystallization, kinetics

## Abstract

Due to its excellent chemical and temperature resistances, short glass fiber reinforced poly (ether ether ketone) composite (SGF/PEEK) is a promising material for application in automotive lightweight. Processing conditions, such as cooling rate, need to be well controlled to obtain the optimal crystallite morphology of PEEK composites. Thus, in this paper, the non-isothermal crystallization kinetics and melting behavior of SGF/PEEK were investigated by differential scanning calorimetry (DSC) at different cooling rates, and the crystallite sizes were evaluated by the X-ray diffraction technique (XRD). Crystallization kinetics models and effective activation energies were evaluated to determine the crystallization parameters of the composites. The results suggest that a lower cooling rate enlarges the size of crystallites and enhances the uniformity of size distribution. The addition of glass fibers improves the nucleation rate owing to heterogeneous nucleation while decreasing the growth rate due to retarded movement of the polymer chain. The combined Avrami-Ozawa equation was shown to describe accurately the non-isothermal crystallization. The absolute value of the crystallization activation energy for SGF/PEEK is lower than that of pure PEEK.

## 1. Introduction

Poly (ether ether ketone) (PEEK) is a type of high-performance engineering plastic with excellent chemical and high temperature resistance [1,2]. Since pure PEEK has a limited performance [3,4], many fiber and nano-particle reinforced PEEK composites have been developed to further improve the mechanical performance. Typically, SGF/PEEK composite has been manufactured with the injection molding process [5,6], and it presents enhanced properties including strength, modulus, rigidity, creep resistance, as well as dimensional and thermal stability [7]. Simultaneously, its density is 30% lower than those of alloys and the cost is reduced by 90% [8]. Therefore, straightforward, application of SGF/PEEK composites for vehicles structural parts has attracted growing interest in vehicle light weighting to accommodate energy saving and emission reducing strategies [9].

It is well known that PEEK is a low crystallinity degree polymer and its properties highly depend on crystallinity and molecular weight [10,11]. The better understanding of the crystallization behavior will allow further optimization of the crystallinity and crystalline structure, which will in turn influence the mechanical properties of PEEK matrix composites [12]. Owing to PEEK’s high melting temperature, the treatments are always high-demanding, many researches have been conducted to confirm the effect of the thermal history on PEEK’s crystallinity and crystallization behavior [13,14]. Cooling condition is a very important factor which can control crystallization behavior and crystal morphology. PEEK can exhibit a double melting peak when crystallization occurs at low cooling rates [13]. The major melting peak corresponds to the classical theory of nucleation and subsequent crystal growth [15]. The secondary melting peak can be ascribed to a combination of simultaneous partial melting and recrystallization of the polymer crystal domains according to the most acknowledged theory at present [16]. There are some researches which have used many kinds of techniques such as DSC, FTIR (Fourier transform infrared spectroscopy), SAXS/WAX (Small-angle X-ray diffraction/Wide-angle X-ray diffraction), to support this interpretation [17,18].

To control the rate and the degree of crystallization, many efforts have contributed to exploring crystallization kinetics. Despite the treatment of non-isothermal crystallization being more complicated, the corresponding research has particular significance, owing to the non-isothermal crystallization being more consistent with practical industrial processing. Also, the non-isothermal crystallization kinetics of PEEK and other PEEK matrix composites can offer great potential for rapid processing in industrial production. Therefore, various works have reported the non-isothermal crystallization behaviors of either pure PEEK or carbon fiber reinforced, nano-particles filled PEEK composites [12,13,14,19,20,21,22,23,24], while the corresponding studies on SGF/PEEK have not been reported. For instance, Cebe developed isothermal crystallization models of PEEK to apply non-isothermal conditions by use of a Hoffman Lauritzen approach based on Velisaris’ method [25,26]. Seo et al. derived a new kinetic model for non-isothermal crystallization and explored the possibility of its application to PEEK [19,27]. Bessard et al. explored the non-isothermal crystallization model of pure PEEK [13]. Similarly, there are some researches on the non-isothermal crystallization kinetics of carbon fiber reinforced PEEK composites [12,21]. The Avrami, Ozawa, and combined Avrami–Ozawa equations have been applied to describe the crystallization kinetics of the alumina-filled PEEK nano-composites by M.C. Kuo et al. [14]. However, the non-isothermal crystallization kinetics of SGF/PEEK composites have not been clearly revealed until now.

Notably, SGF /PEEK composites can be used in large numbers of structural and functional parts [28]. To optimize processing conditions and minimize processing time, it is significant to understand the crystallization kinetics for SGF/PEEK composites under different cooling rates and to identify the effects of processing conditions on the ultimate microstructure and properties. Besides, in order to obtain excellent mechanical properties of SGF/PEEK, it is necessary to study the crystallization behavior and morphology in advance. Obviously, different PEEK composites require specific models to analyze their crystallization behaviors [3,14,20,25,29,30]. However, up to now, suitable non-isothermal crystallization models for SGF/PEEK have not been reported.

Thus, this work aims to investigate the influence of the addition of glass fibers on the non-isothermal crystallization behavior of SGF/PEEK under different cooling conditions. The kinetic parameters of pure PEEK and SGF/PEEK composites were obtained by differential scanning calorimetry (DSC), and the cooling rate corresponding to the narrowest size distribution of crystallite was determined. X-ray diffraction (XRD) was applied to study the morphology and size of crystallite. Modified Avrami, Ozawa, and Ozawa–Avrami equations were applied to make a linear fitting to explore the crystallization kinetics. All results acquired from the SGF/PEEK composites were compared with those of pure PEEK to evaluate any changes in crystallization. The consequences acquired can provide a theoretical foundation to guide the molding process and regulate the properties of PEEK composites.

## 2. Experimental

### 2.1. Materials

The material studied here is a type of short glass fiber reinforced PEEK composite (SGF/PEEK). Besides, pure PEEK was also studied for the purpose of contrast. The PEEK composite with 30 wt % glass fiber, was supplied by Jilin Zhongyan Special Plastic Co. Ltd (Changchun, China), the length of the glass fiber being 3–6 mm. The pure PEEK with a weight-average molecular weight ($M_{w}$) of 10^5^ g/mol, was provided by Jiangsu Junhua Special Plastic Co. Ltd. (Changzhou, China).

### 2.2. Characterization Method

Non-isothermal crystallization analyses were conducted by differential scanning calorimeter (DSC214, NETZSCH, Selb, Bavaria, Germany). All experiments were conducted under a nitrogen atmosphere at a flow rate of 40 mL/min to avoid oxidation. The weight of per sample was between 8 mg and 12 mg. First, SGF/PEEK samples were heated to 380 °C at a rate of 40 °C/min and held at 380 °C for 5 min to erase thermal history, then cooled down to room temperature at cooling rates of 5, 10, 20, 40 °C/min, respectively. Finally, the sample was heated to 380 °C at 10 °C/min again. The same operations were also applied to all pure PEEK samples. X-ray diffraction analysis (SIMENS D500, Munich, Germany) recorded with a Cu K_α_ radiation (40 kV, and 30 mA) at a step size of 0.05° and a step time of 1 s, was employed to analyze the crystalline morphology of SGF/PEEK composites at different cooling rates (5, 10, 20, and 40 °C/min). Crystallite size ($L_{hkl}$, in Å unit) was obtained from peak broadening from the Scherrer equation [12]:

(1) $$L_{hkl}=K\lambda /\cos\theta \beta_{0}$$

(2) $${\beta_{0}}^{2}=\beta_{M}^{2}-\beta_{I}^{2}$$

where $\beta_{0}$ is the width of the diffraction beam (rad); $\beta_{M}$ is the measured width of the diffraction beam (rad); $\beta_{I}$ is the instrument width (rad); *K* is the shape factor of crystalline thickness, relating to $\beta_{0}$ and $L_{hkl}$ when $\beta_{0}$ is defined as the half-height width of diffraction peaks, *K* = 0.89.

## 3. Results and Discussion

### 3.1. Non-Isothermal Crystallization Behavior

**Figure 1:**
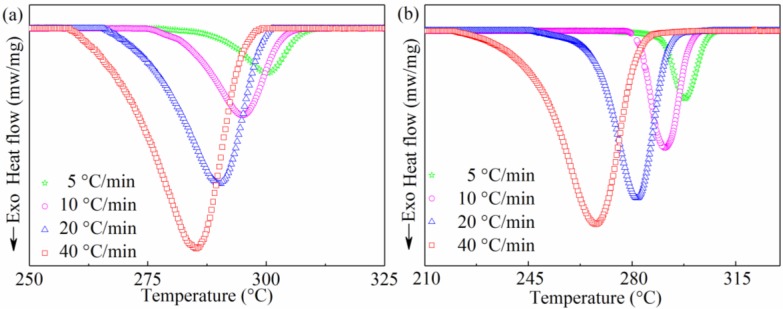
DSC thermograms of non-isothermal crystallization for all samples: (**a**) pure poly (ether ether ketone) (PEEK), (**b**) short glass fiber reinforced poly (ether ether ketone) composite (SGF/PEEK) under different cooling conditions.

Typical DSC curves of the non-isothermal crystallization exotherm for the SGF/PEEK and pure PEEK samples under various cooling conditions are shown in Figure 1. The DSC crystallization parameters, such as the onset crystallization temperature ($T_{o}$), crystallization peak temperature ($T_{p}$), the width at half-height of the exotherm peak (ΔW), and the crystallization enthalpy ($\Delta H_{c}$) measured from the non-isothermal crystallization exotherms are summarized in Table 1. Obviously, for all samples, $T_{o}$ shifts to lower temperature with the increasing cooling rate. It illustrates that crystallization will occur at lower temperature at a higher cooling rate. Similarly, $T_{p},$ which implies the maximum crystallization rate, decreases. Actually, it can be attributed to the less time required to activate nuclei to crystallize with the increasing cooling rate. The polymer needs the more super-cooled region ($\Delta T=T_{m}-T_{p}$) to activate the crystallization process. The results are consistent with the results of alumina nanoparticle filled poly(ether ether ketone) and PEEK/SCF/nano-SiO2 composites [14,21]. It was found that two paradoxical effects occur simultaneously when the SGF/PEEK composite undergoes crystallization [12]. On the one hand, retarding the movement of the polymer chain results in a negative effect on the crystallite, then, $T_{o}$ and $T_{p}$ decrease. On the other hand, the heterogeneous nucleation can accelerate the deposition of polymer molecules which will increase $T_{o}$ and $T_{p}$ [12]. When adding 30 wt % GF to PEEK, the $T_{p}$ of the SGF/PEEK was found to be lower than that of the pure PEEK. It is very clear that the combination of 30 wt % GF shifts the main mechanism from offering more spaces for nucleation to retarding the folding adjustment of the polymer chain. In conclusion, the GF in the PEEK matrix retards the mobility of the polymer chain, and in turn smaller spherulites with more defects are obtained. This differs from the homogeneous crystallization of pure PEEK.

We can also find that ΔW and $\Delta H_{c}$ increase as the cooling rate increases. At the cooling rate of 5 °C/min, the most uniform size distribution of crystallite and minimum crystallization enthalpy are obtained, respectively. Actually, the lower value of $\Delta H_{c}$ implies that crystal growth during the non-isothermal crystallization process is easier.

The half time of crystallization ($t_{1/2}$) is the time to achieve 50% relative crystallinity. The entire crystallization time ($t_{c}$) is a significant factor in making a description of the non-isothermal crystallization rate and is defined with the following equation:

(3) $$t_{c}=\left|T_{e}-T_{o}\right|/\Phi$$

where $T_{o}$ and $T_{e}$ are the onset and end temperatures of crystallization, respectively and Φ is the cooling rate. A shorter crystallization time is a result of a smaller crystallite size. The crystallization time notably decreased as the cooling rate increases, revealing that there is less time available to develop perfect crystallites.

According to the DSC measurements, the absolute crystallinity fraction $X_{c}$ of SGF/PEEK under different cooling rates can be expressed by relating to the heat of fusion of an infinitely thick PEEK crystal [31]:

(4) $$X_{c}=\Delta H_{c}/(\Delta H_{f}^{0}W_{polymer})\cdot 100\%$$

where $\Delta H_{f}^{0}$ is a reference value that represents the heat of fusion of a pure crystalline polymer. For PEEK, $\Delta H_{f}^{0}$ is 130 J/g. $W_{polymer}$ is the weight fraction of polymer matrix. The values for $X_{c}$ of all samples under different cooling conditions are presented in Table 1. Since molecular chains have adequate time for rearrangement, the low cooling rate generates a high degree of crystallinity. Moreover, the combination of GF would particularly bring about lower crystallinity fractions of the SGF/PEEK composites, as compared with the pure PEEK.

As a function of temperature, the relative crystallinity, $X_{c}\left(T\right)$, can be expressed with the following equation [32]:

(5) $$X_{c}\left(T\right)=\int_{T_{o}}^{T}\left(dH_{c}/dT\right)dT/\int_{T_{o}}^{T_{e}}\left(dH_{c}/dT\right)dT$$

where $T_{o}$ and $T_{e}$ are the temperatures of onset and end crystallization, respectively. *T* is the experimental temperature, and $dH_{c}$ is the heat flow rate.

In non-isothermal crystallization, the temperature can be replaced by time according to equation [33]:

(6) $$t=(T_{o}-T)/\Phi$$

where $T_{o}$ is the onset crystallization temperature, *T* is the crystallization temperature at time t, and Φ is the cooling rate.

It is proposed that the relative crystallinity as a function of time ($X_{c}\left(t\right)$) can be defined as:

(7) $$X_{c}\left(t\right)=\int_{t_{o}}^{t}\left(dH/dt\right)dt/\int_{t_{o}}^{t_{e}}\left(dH/dt\right)dt$$

where $t_{o}$ and $t_{e}$ are on behalf of onset crystallization time and end crystallization time and H is heat flow, and t represents time during the course of crystallization.

**Figure 2:**
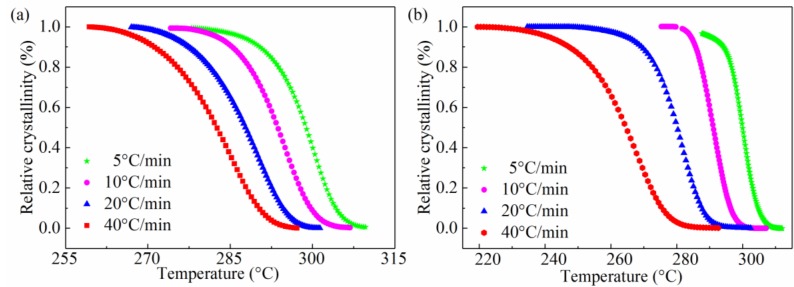
Relative crystallinity versus temperature for all samples: (**a**) pure PEEK, (**b**) SGF/PEEK.

**Figure 3:**
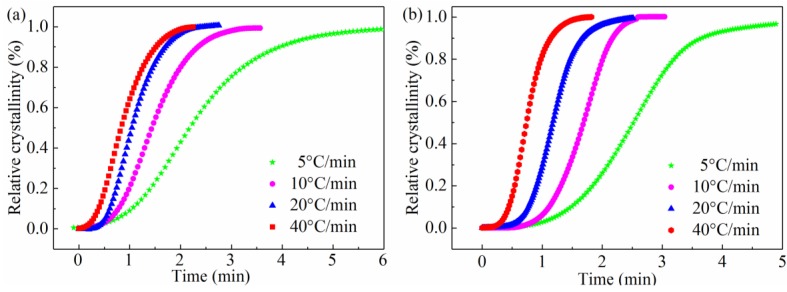
$X_{c}\left(t\right)$ vs. time for all samples: (**a**) pure PEEK and (**b**) SGF/PEEK.

According to the Equations (5) and (7), we plot Figure 2 and Figure 3. They indicate that the relative crystallinity of all samples is a function of temperature and time. It reveals that the shape of all plots is sigmoidal in Figure 2, and the curvature of the upper parts of curves could be attributed to the spherulites impingement in the secondary crystallization. We can find that the shape of the plot for all samples in Figure 3 is reverse to Figure 2 and crystallization time is reduced as cooling rate increases. This makes it clear that the SGF/PEEK composites have not sufficient time for crystallizing at a higher cooling rate and as a result, smaller crystals with more defects exist.

### 3.2. Crystalline Melting Analysis

**Figure 4:**
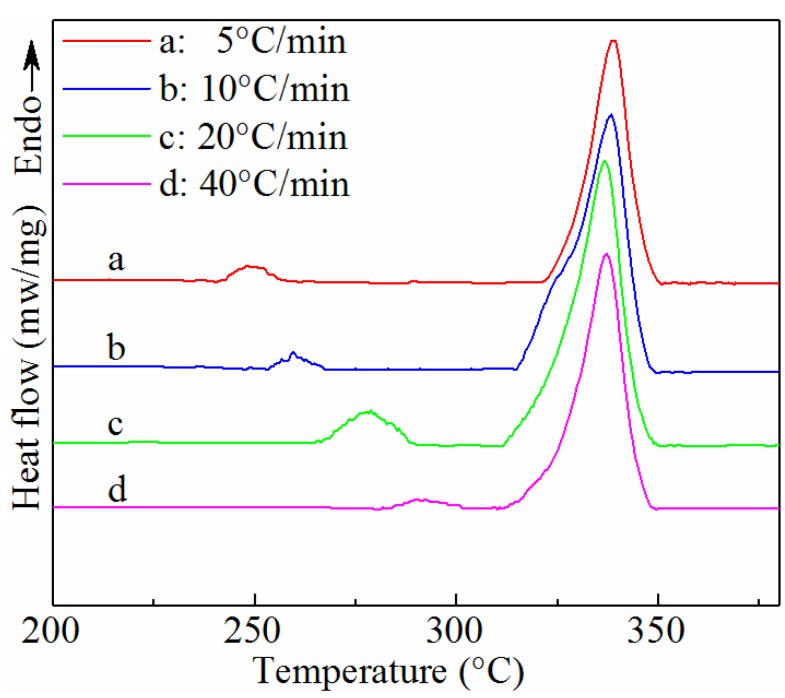
DSC melting curves of non-isothermal crystalized SGF/PEEK at different cooling rates.

The melting behavior for SGF/PEEK samples can be further verified via the DSC melting endotherms, recorded after non-isothermal crystallization at different cooling rates in Figure 4. The DSC endotherms show a double-melting behavior, which seems much more obvious for the SGF/PEEK samples than that of pure PEEK. The double-melting behaviors of pure PEEK and SGF/PEEK can both be almost ignored for higher cooling rates [12]. The main peak noticed at an identical temperature of 343 °C is assigned as $T_{m}$. On the melting curves of the slowly cooled samples (≤20 °C/min), another peak is observed in the early stage of fusion and shows that mechanisms of secondary crystallization appear. As shown in Table 2, the lower melting temperature ($T_{m1}$) changes as the cooling rate increases for all samples. Conversely, the temperature ($T_{m2}$) and shape of the main upper peak remain constant as a result of the different cooling treatment. As shown in Figure 4, we can draw the conclusion that with increasing cooling rate, the lower endothermic peak always moves to a higher temperature with a larger enthalpy. Accordingly, it remains metastable or at non-equilibrium. Two contradictory explanations of the sample’s double melting peak can be discovered in documented research: some literatures support that the two endotherms symbolize the melting of two morphologically disparate crystallite groups developed during the crystallization process [34,35], nevertheless, the vast majority of latest researches ascribed the multiple melting peaks to a combination of a melting–recrystallization–remelting course of the primitive crystallites [16,17,36].

Lee and Porter assumed that the lower melting peak is due to partial melting of the original crystals: partial melting followed by a recrystallization course is deemed to occur at crystal surfaces [37]. Meanwhile, the major melting peak represents the melting of reorganized crystallites in the process of heating. Those crystallized at high cooling rates show a smaller endothermic peak at higher $T_{m1}$, due to a smaller quantity of secondary crystals developed in the course of crystallization.

### 3.3. X-Ray Diffraction (XRD) Analysis

**Figure 5:**
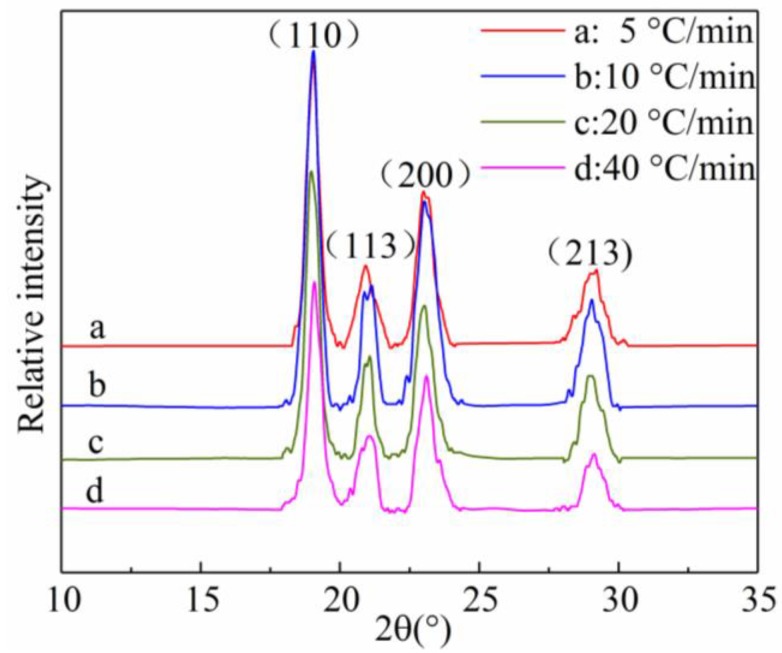
XRD patterns of SGF/PEEK samples at different cooling rates.

The X-ray diffraction patterns of the samples obtained are shown in Figure 5. The characteristic peaks of SGF/PEEK at the scattering diffraction angles 2θ of 19.05°, 21.09°, 23.07°, and 29.17° can be ascribed to the lattice planes (110), (113), (200), (213), respectively, and they also exist in all the analyzed samples. No new peak appears, suggesting that no noticeable reaction occurs between PEEK and GF during the melting and crystallization processes. Meanwhile, the broadened peak would be a result of a smaller size of crystallite being developed during the crystallization phases or attributable to structural defects. The X-ray diffraction results for all the samples were similar under the various cooling rates, which means that the dimensions of the PEEK crystal cell did not change while, the intensity of the peaks have some subtle changes, suggesting some variations in crystallinity. The higher intensity represents a higher level of crystallinity. This was further researched by DSC measurement in Figure 1.

**Figure 6:**
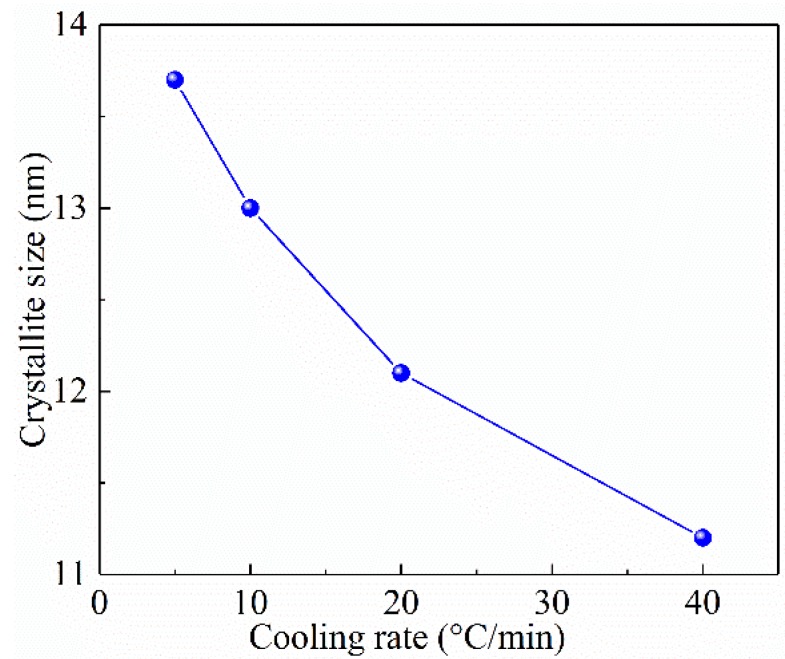
Data obtained by XRD patterns: average crystallite size vs. cooling rate.

So as to demonstrate the influence of cooling rates on the crystallite size, the trends of average crystallite size corresponding to the cooling rate can be obtained from the XRD patterns (Figure 6). The average size of crystallite decreases gradually with the cooling rate due to the poor diffusion rate of PEEK polymer chains as the cooling rate increases.

Based on the rules reported in references [38,39,40,41], we can reasonably predict that for the purpose of achieving high mechanical strengths of SGF/PEEK, high cooling rates should be used, leading to a relatively small crystallite size. On the contrary, in order to obtain high stiffness, low cooling rates should be used to form a large crystallite size in SGF/PEEK.

### 3.4. Non-Isothermal Crystallization Kinetics

#### 3.4.1. Modified Avrami Equation

Under isothermal crystallization conditions, the Avrami approach gives a description of the crystal growth by n and $Z_{t}$ coefficients [42]. $Z_{t}$ is the crystallization rate constant, decided by temperature, *n* is the Avrami exponent which offers information on nucleation and growth geometry. In Equations (8) and (9), $X_{c}\left(t\right)$ is the relative degree of crystallinity with respect to time *t*:

(8) $$1-X_{c}\left(t\right)=\exp\left(-Z_{t}t^{n}\right)$$

(9) $$\log[-ln(1-X_{c}\left(t\right))]=n\log t+\log Z_{t},$$

It is well known that the equation is mainly used to depict isothermal conditions. When converted into non-isothermal conditions, it can only interpret the initial crystallization phase. For the purpose of modifying the Avrami equation, the effectiveness of the crystallization rate ought to be taken into account. In consequence, the value $Z_{t}$ should be replaced with $Z_{c}$ as follows [14]:

(10) $$\log Z_{c}=\log Z_{t}/\Phi$$

where Φ is the cooling rate.

**Figure 7:**
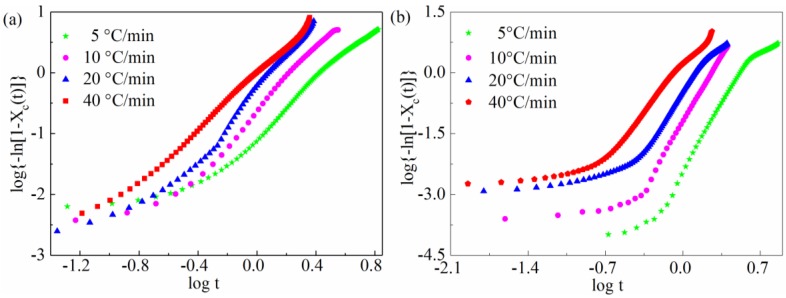
Modified Avrami plots of $\log\left[-\ln\left(1-X_{c}\left(t\right)\right)\right]$ versus log*t* at various cooling rates for non-isothermal crystallization: (**a**) pure PEEK and, (**b**) SGF/PEEK.

As shown in Figure 7, the curve of $\log\left[-\ln\left(1-X_{c}\left(t\right)\right)\right]$ with respect to logt presents the slope n and the intercept $Z_{c}$. Table 3 gives the Avrami parameters n and $Z_{c}$ for all samples under non-isothermal crystallization. The average value of the Avrami exponent n for pure PEEK is approximately 2.56. In addition, the n values of the SGF/PEEK composites are higher than those of pure PEEK, reaching 4.72 at a cooling rate of 10 °C/min. We conclude that the nucleation mechanism and the growing geometry of the PEEK chain in SGF/PEEK appear to be more complicated [43]. Actually, the n value decreases implying that less crystallization time will restrict the growth of polymer crystallites and conduct simpler geometry of crystallites, which will conversely cause the lower value of the Avrami exponent. Meanwhile, the $Z_{c}$ lowering may due to the mobility hindrance of the PEEK polymer chain. The $Z_{c}$ values for the SGF/PEEK composites are lower than those of the pure PEEK. Consequently, the inclusion of the glass fiber may retard the non-isothermal crystallization, but offer more sites for crystallization.

In summary, the modified Avrami equation was used mainly for the primary crystallization at which the complicated impingement effect does not occur since the continuous decrease in the cooling rate is not considered.

#### 3.4.2. Ozawa Equation

On the basis of the Ozawa equation [14], there is an assumption that non-isothermal crystallization is constituted by infinitesimal isothermal steps, and is expressed as:

(11) $$1-X_{c}\left(T\right)=\exp\left(-K\left(T\right)/{\left|\Phi \right|}^{m}\right)$$

(12) $$\log[-\ln(1-X_{c}\left(T\right))]=\log\left(K\left(T\right)\right)-m\log\Phi$$

where $X_{c}\left(T\right)$ is the relative degree of crystallization with regard to temperature T, and K(T) is named as the cooling function and depicts the crystallization rate which is merely temperature dependent. Parameter m is determined by the nucleation behavior and crystal growth mechanism which is for the dimensions of crystal growth.

**Figure 8:**
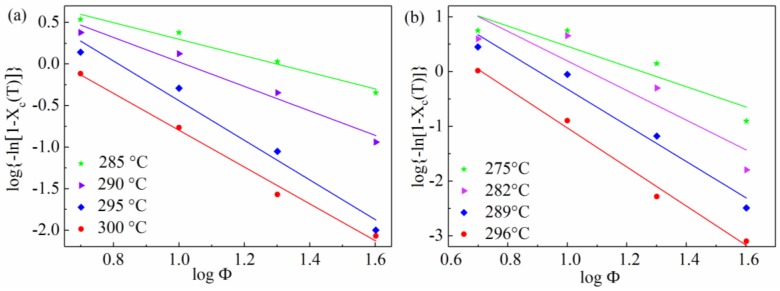
Ozawa plots of $\log\left[-\ln\left(1-X_{c}\left(T\right)\right)\right]$ vs. logΦ for non-isothermal crystallization of all samples: (**a**) pure PEEK, (**b**) SGF/PEEK.

The plot of $\log\left[-\ln\left(1-X_{c}\left(T\right)\right)\right]$ against logΦ is depicted in Figure 8, and the slope and intercept give the Ozawa exponent m and K(T). Under non-isothermal crystallization, the crystallization rate is still not constant but is a function of both time and cooling rate. Nevertheless, these differences need not be considered in the Ozawa model. Under different cooling rates, the slow secondary crystallization was not taken into account, which may decrease m and K(T) as Alan J. Lesser et al. stated [44]. This equation also overlooks another parameter, which is the folded chain length of the polymer. It is a function of the crystallization temperature, illustrating that the different folded chain lengths will lead to dynamic crystallization. 

All the above explains why the Ozawa model is not perfectly successful in giving a description of the crystallization behavior of SGF/PEEK polymers.

#### 3.4.3. Combined Avrami-Ozawa Equation.

From the above analysis it could be seen that the two models mentioned could not accurately express the non-isothermal crystallization kinetics of the SGF/PEEK composites. Mo et al. propose a combined Avrami-Ozawa equation to describe the dynamic crystallization behavior [45], as follows:

(13) $$\log\left(Z_{t}\right)+n\log\left(t\right)=\log\left(K\left(T\right)\right)-m\log\left(\Phi \right)$$

(14) log(Φ)=log(F(T))−blog(t)

(15) $$F\left(T\right)={\left(K\left(T\right)/Z_{t}\right)}^{\frac{1}{m}}$$

(16) b=n/m

**Figure 9:**
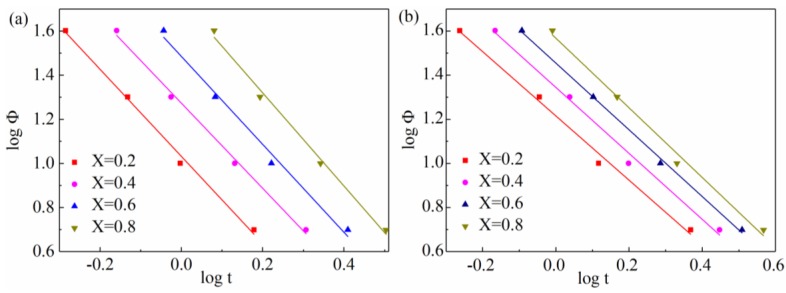
Avrami–Ozawa plots of logΦ vs. log*t* for non-isothermal crystallization of all samples: (**a**) pure PEEK, (**b**) SGF/PEEK.

The combined Avrami-Ozawa model gives a description of the relationship between the crystallization time t and the cooling rate Φ for a given crystallinity under dynamic crystallization. In theory, the physical meaning of F(T) is the necessary value of cooling rate approaching a given crystallinity at unit crystallization time [45]. The plot of logΦ versus logt will calculate the slope and intercept of *b* and log(F(T)), respectively. In Figure 9, there is a good linear relationship between logΦ and logt (correlation coefficient *R*^2^ > 0.99, which is higher than those of modified Avrami and Ozawa equation fittings). This shows that the combined Avrami–Ozawa equation can accurately confirm the non-isothermal crystallization behaviors of PEEK and SGF/PEEK composites.

As listed in Table 4, F(T) shows a steady increase as the relative crystallinities increase, suggesting that, at unit crystallization time, a higher cooling rate is needed so as to achieve a higher crystallinity degree [21]. The larger temperature span would cause crystallization to occur with a smaller crystallite size at a given relative crystallinity. Accordingly, the higher F(T) might account for the larger super-cooled region ($T=T_{m}-T_{p}$) during the non-isothermal crystallization phase. The higher ΔT might be necessary for more hindrance of mobility.

The slope *b* of the SGF/PEEK composites shows very subtle changes at the initial crystallization phases of which the relative crystallinity is lower than sixty percent, while it increases in the following process, and the phenomenon is not affected by the GF reinforcement. It demonstrates that there exists a slow secondary crystallization regime at a high degree of relative crystallinity.

#### 3.4.4. Crystallization Active Energy

The Friedman approach better fits the non-isothermal crystallization research [46]. Accordingly, the differential iso-conversional method of Friedman was used in this paper. For a given process, the kinetic equation of this approach can be expressed as:

(17) dα/dt=K(T)f(α)

where K(T) is the Arrhenius constant, α is the conversion degree, and f(α) is the function representing the reaction mechanism. When analyzing DSC data, the conversion degree may be defined as:

(18) α=H/Q

where H is the amount of heat involved in a reaction at a conversion degree α and Q is the total amount of heat involved in the overall reaction. The kinetic equation, in the logarithmic form, hence can be written as:

(19) $$\ln(d\alpha /dt)=\ln\left(A_{\alpha }f\left(\alpha \right)\right)-E_{\alpha }/(RT_{\alpha })$$

where $A_{\alpha }$, $E_{\alpha }$, $T_{\alpha },$ and R are the pre-exponential parameter, the effective active energy, the temperature at a specific conversion degree α, and the gas constant (*R* = 8.3145 J/mol/K), respectively.

**Figure 10:**
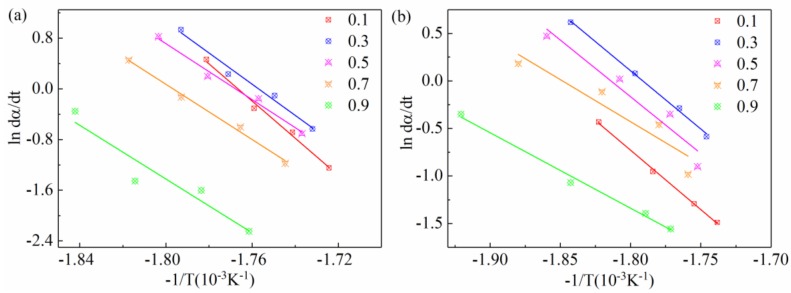
Friedman plots of lndα/dt vs. −1/*T* for non-isothermal crystallization for all samples: (**a**) pure PEEK, (**b**) SGF/PEEK at various cooling rates.

By plotting the ln(dα/dt) with respect to −1/T curves for all samples (Figure 10), the corresponding active energies (in Table 5) could be obtained from the slope which is equal to $-E_{a}/R$ under the range of degrees of crystallinity (i.e., from 10% to 90%).

The short glass fibers also have two major effects on the crystallization process of polymer [47]. On the one hand, short glass fiber acts as heterogeneous nuclei to induce polymer chains to crystallize on their surfaces. On the other hand, the binding force between the nucleating agent and polymer melt is very weak, and therefore blocks the polymer mobility. The $E_{\alpha }$ of SGF/PEEK samples vary from −103.89 to −64.69 kJ/mol. The $E_{\alpha }$ of pure PEEK varies from −320.03 to −287.59 kJ/mol. Meanwhile, the absolute values of effective activation energy of SGF/PEEK are lower than those of pure PEEK, illustrating that the 30 wt % glass fiber reduces the nucleation energy barrier to promote crystal growth.

## 4. Conclusions

This study systematically investigated the non-isothermal crystallization and melting behavior of SGF/PEEK. Compared with pure PEEK, glass fiber, which acts as a heterogeneous agent, remarkably affects the crystallization behavior of PEEK matrix in SGF/PEEK composites. The main conclusions are as follows:

The crystallization parameters of SGF/PEEK, such as the crystallization peak temperature $T_{p}$ and the absolute degree of crystallinity fraction $X_{c}$ are lower than those of pure PEEK at the same cooling rate. The reason is ascribed to the fact that GF could retard the mobility of the polymer chains. Specifically, by adding the glass fiber, the overall crystallization time of SGF/PEEK can be reduced due to the combined effects of rapid heterogeneous nucleation and the short time to grow to the small final crystal grain size. Besides, the lower cooling rate generates a narrower size distribution of crystallite (ΔW), while the average crystallite size tends to decrease gradually with increasing cooling rate.

By comparing different crystallization kinetics for all samples, we found that the combined Avrami–Ozawa method can describe well non-isothermal crystallization. Moreover, the value of F(T) increases for all samples, suggesting that with unit crystallization time, a higher cooling rate is needed to obtain a higher crystallinity. Besides, the Avrami exponent values of the SGF/PEEK composites are higher than those of the pure PEEK, indicating that the glass fibers imparted a more complex geometry effect on the crystallization of the PEEK matrix. The values of parameter *b* vary with cooling rates, revealing that mechanism of SGF/PEEK crystallization is in a pronounced way affected by both cooling conditions and the addition of glass fiber. The addition of 30 wt % GF greatly reduced the absolute value of the crystallization activation energy of PEEK due to the reduced nucleation energy barrier. 

All results demonstrate that processing conditions such as cooling conditions need to be chosen exactly to guarantee the desired crystallite morphology, and this can offer a theoretical basis for the optimal performances of PEEK composites. 

## Figures and Tables

**Table 1:** The non-isothermal crystallization parameters determined from DSC exotherms for all samples.

Samples	Φ (°C/min)	$T_{o}$ (°C)	$T_{p}$ (°C)	$t_{c}$ (min)	$1/t_{1/2}$ min^−1^	ΔW	$\Delta H_{c}$ (J/g)	$X_{c}$ (%)
PEEK	5	307.91	300.57	3.90	0.44	10.67	−51.95	39.96
	10	303.78	295.30	2.21	0.70	12.06	−49.89	38.38
	20	299.54	290.86	1.38	0.96	14.29	−47.76	36.74
	40	295.51	285.12	0.77	1.23	17.10	−43.55	33.50
SGF/PEEK	5	306.45	297.63	3.26	0.61	9.46	−26.99	29.66
	10	300.35	291.21	1.86	1.10	10.49	−26.21	28.80
	20	292.89	281.71	1.36	1.45	14.20	−26.09	28.67
	40	283.16	268.11	0.99	2.00	21.03	−25.74	28.29

**Table 2:** Melting parameters obtained after non-isothermal crystallization for SGF/PEEK.

Samples	Φ (°C/min)	$T_{m1}$ (°C)	$T_{m2}$ (°C)
	5	248.88	338.71
SGF/PEEK	10	259.67	338.02
	20	278.47	336.62
	40	292.05	336.99

**Table 3:** Calculated n and $Z_{c}$, values at different cooling rates for all samples.

Cooling Rate (°C/min)	PEEK		SGF/PEEK	
	n	$Z_{c}$	n	$Z_{c}$
5	2.29	0.62	4.65	0.33
10	2.73	0.86	4.72	0.76
20	2.95	0.97	4.17	0.89
40	2.25	1.00	3.46	1.02

**Table 4:** Calculated b and logF(T) values at different cooling rates for a given relative crystallinity.

$X_{c}\left(t\right)$	PEEK		SGF/PEEK	
	b	F(T)	b	F(T)
20%	1.97	10.72	1.46	16.60
40%	1.93	18.62	1.49	21.88
60%	1.99	30.20	1.51	28.18
80%	2.11	54.95	1.58	37.15

**Table 5:** Crystallization activation energies as a function of the conversion degree α.

Conversion Degree α	SGF/PEEK	PEEK
	$E_{\alpha }$ (KJ/mol)	$E_{\alpha }$ (KJ/mol)
0.1	−103.89	−320.03
0.3	−101.88	−309.50
0.5	−99.18	−301.45
0.7	−73.63	−291.01
0.9	−64.69	−287.59
